# Smectite clay minerals reduce the acute toxicity of quaternary alkylammonium compounds towards potentially pathogenic bacterial taxa present in manure and soil

**DOI:** 10.1038/s41598-020-71720-5

**Published:** 2020-09-21

**Authors:** Benjamin Justus Heyde, Stefanie P. Glaeser, Linda Bisping, Kristin Kirchberg, Rüdiger Ellinghaus, Jan Siemens, Ines Mulder

**Affiliations:** 1 Institute of Soil Sciences and Soil Conservation, iFZ Research Centre for Biosystems, Land Use and Nutrition, Justus Liebig University, Heinrich-Buff-Ring 26-32, 35392 Gießen, Germany; 2 Institute for Applied Microbiology, iFZ Research Centre for Biosystems, Land Use and Nutrition, Justus Liebig University, Heinrich-Buff-Ring 26-32, 35392 Gießen, Germany; 3 Institute of Physical Chemistry, Justus Liebig University, Heinrich-Buff-Ring 17, 35392 Gießen, Germany

**Keywords:** Environmental sciences, Environmental impact

## Abstract

Quaternary alkylammonium compounds (QAACs) are a group of cationic surfactants which are disinfectants with numerous industrial and agricultural applications and frequently released into the environment. One recent hypothesis is that bacteria present in soil will be protected from acute toxic effects of QAACs in the presence of expandable layer silicates due to interlayer sorption. We therefore studied bacterial growth kinetics with high temporal resolution and determined minimal inhibitory concentrations (MICs) of two QAACs, benzyldimethyldodecylammonium chloride (BAC-C12) and didecyldimethylammonium chlorid (DADMAC-C10), for eight strains of different bacterial taxa (*Escherichia coli, Acinetobacter*, *Enterococcus faecium*, *Enterococcus faecalis,* and *Pseudomonas fluorescens*) in relation to QAAC sorption to smectite and kaolinite. The MICs of BAC-C12 and DADMAC-C10 were in the absence of smectite and kaolinite in the order of 10 to 30 µg mL^−1^ and 1.0 to 3.5 µg mL^−1^ for all strains except the more sensitive *Acinetobacter* strain. For all tested strains and both tested QAACs, the presence of smectite increased apparent MIC values while kaolinite had no effect on MICs. Sorption curves without bacteria showed that smectite sorbed larger amounts of QAACs than kaolinite. Correcting nominal QAAC concentrations employed in toxicity tests for QAAC sorption using the sorption curves explained well the observed shifts in apparent MICs. Transmission electron microscopy (TEM) demonstrated that the interlayer space of smectite expanded from 13.7 ± 1 Å to 19.9 ± 1.5 Å after addition of BAC-C12. This study provides first evidence that low charge 2:1 expandable layer silicates can play an important role for buffering QAAC toxicity in soils.

## Introduction

The 2009 OECD list of High Production Volume Chemicals (HPVC) catalogues those chemical substances with annual production exceeding 1000 Mg in at least one OECD member country “in order to identify those which are potentially hazardous to the environment and/or to the health of the general public or worker”^1^. Surfactants are an important class of HPVCs and several neutral and anionic surfactants have extensively been investigated in various environmental compartments. However, the number of studies concerned with the transport, fate, and possible adverse effects of cationic surfactants in the environment is small, despite the fact that electrostatic and hydrophobic interactions with negatively charged surfaces in soil and sediments could promote their persistence^2^. Within the group of cationic surfactants, quaternary alkylammonium compounds (QAACs) are a heterogeneous group of organic compounds comprising a quaternary nitrogen atom. This nitrogen atom carries one permanent positive charge and at least one alkyl chain rest^3^, which together cause their amphiphilic properties used for disinfection and sanitation purposes. The antimicrobial activity of QAACs is mainly based on the interaction of QAAC molecules with cell membranes due to the positively charged nitrogen atom, causing disturbance of membrane integrity and subsequent leakage of cellular contents^4^.

Production and consumption data for these QAACs are scattered, but volumes that are released into the environment are tentatively orders of magnitude higher than is the case for example for pharmaceuticals^2^. For agricultural soils, direct application of QAACs with manure, sewage sludge, during irrigation with wastewater or as biocides or adjuvants in agricultural pesticide formulations are the main entry pathways. The use of QAACs as adjuvants for pesticide application enhances solubility of poorly soluble pesticide compounds, rain fastness and penetration of pesticides. Tezel^5^ provided consumption data for the biocidal adjuvants in the State of California for BAC-C12-16, DADMAC-C10, DADMAC-C8-10, and DADMAC-C8, totaling about 5000 kg in one year. Typical QAAC concentrations in agrochemical tank-mixed sprays range from 0.05 to 0.5% v/v^6^. According to Mulder et al.^2^ predicted environmental concentrations (PEC) for QAACs that are applied to soils with manure are in the order of 3.5 mg kg^−1^ and the median PEC of QAACs in sewage sludge amended soils was reported to be 25 µg kg^−1^. In a screening study on Swedish sewage sludge and wastewater, Östman et al.^7^ demonstrated that QAACs, with the exception of metals, are the biocides encountered at highest concentrations. The concentrations determined in sewage sludge were at least one order of magnitude higher than other antimicrobial active substances with average concentrations often exceeding 100 µg g^−1^ d.w.

The release of large quantities of QAACs into the environment poses a risk for environmental and human health especially with regard to the evolution and spreading of disinfectant and antibiotic resistant bacteria. For example, wastewater irrigated agroecosystems that are exposed to high QAAC loads were spotlighted as “hot spots” of selection for antibiotic resistant bacteria^8,9^. Decisive for the evolution and propagation of antibiotic resistant bacteria within these systems is possibly not only the direct selection by antibiotic agents, but also co- and cross-selection by other compounds such as QAACs, which are used in much higher quantities than antibiotic substances^8,10–14^. Gaze et al.^15^ and Tandukar et al.^16^ reported the occurrence of antibiotic and QAAC resistance genes in soil and the alteration of microbial communities as a result of long-term exposure to QAACs. Plasmid and genome sequence based studies showed that several QAAC resistance genes are located on the same genetic units as antibiotic resistance genes, promoting the co-selection of antibiotic resistance genes in the presence of QAACs^15–18^.

The eco-toxicological effects of organic pollutants in the environment depend largely on their bio-accessibility and persistence, both of which are heavily affected by the pollutant’s sorption to constituents of soils and sediments^19^. Due to the molecular nature of QAACs, based on results of sorption studies, and as known from the preparation of organoclays for industrial applications, it appears likely that these compounds are adsorbed and retained by soil clay minerals and soil organic matter. More specifically, in soils, potential adsorbents include soil organic matter, clay minerals, oxides, proteins, and microbial cell walls^2,20^. Sorption of the QAACs can potentially retard their biodegradation and reduce their toxicity, with interlayers of expandable layer silicate minerals appearing particularly suitable to sequester QAACs.

We hypothesized that bacteria exhibit strain specific growth response to QAAC exposure leading to QAAC specific MICs, which are shifted to higher apparent concentrations due to a reduction to acute toxicity as a consequence of QAAC sorption to clay minerals. We further hypothesized that a stronger binding of QAACs to interlayers of expandable smectite clay minerals causes a stronger reduction of toxicity compared to the sorption to non-expandable 1:1 layer kaolinite. In order to test these hypotheses, we determined (1) growth behavior and minimal MICs of BAC-C12 and DADMAC-C10 for a selection of eight bacterial strains including Gram-negative and Gram-positive bacteria representing potential pathogens that can originate in soils for example from the application of manures and (2) investigated the MIC values and sorption curves of BAC-C12 and DADMAC-C10 in the presence of smectite and kaolinite.

## Results

### Strain specific growth response to QAAC exposure and QAAC specific MICs

MIC values for BAC-C12 and DADMAC-C10 were in a similar range of 10 to 30 µg mL^−1^ and 1.0 to 3.5 µg mL^−1^, respectively, for all tested strains according to their compound specific susceptibilities (Table 1). Only the *Acinetobacter* strain showed a higher sensitivity to BAC-C12 with a MIC below 5 µg mL^−1^.

**Table 1:** Overview of minimal inhibitory concentrations (MICs) of BAC-C12 and DADMAC-C10 on the growth of tested reference strains. Values in brackets indicated the number of replicates which shared a respective value.

Strains	MIC BAC-C12	MIC DADMAC-C10
	[µg mL^−1^]	[µg mL^−1^]
*E. coli* ESBL37B15_13_1E	15 (1x)/25 (1x)	3 (1x)/ 3.5 (1x)
*E. coli* ESBL232B15_13_2E	15 (2x)/20 (2x)	2.5 (2x)
*E. coli* ESBL370B15_13_2A	15 (2x)	2.5 (2x)
*E. coli* ConF4	10 (2x)	2/2.5 (3x)
*Acinetobacter* sp. KPC-SM-21	< 5 (2x)	2.5 (2x)
*P. fluorescens* DSM 50090^T^	25 (4x)/30 (1)	3 (3x)/ 3.5 (1)
*E. faecium* DSM 20477^T^	15 (2x)	2 (2x)
*E. faecalis* DSM 20478^T^	15 (2x)	1 (1x)/1.5 (1x)

**Figure 1:**
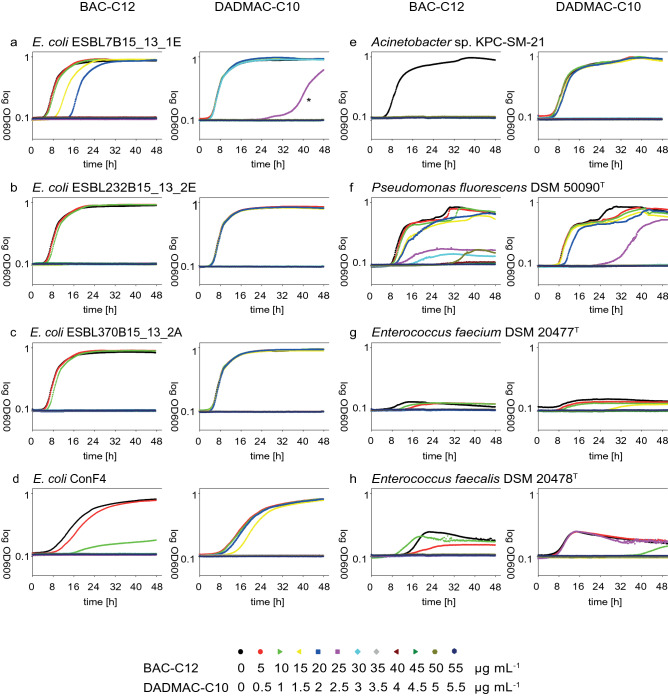
Growth curves for all eight tested bacterial strains in the presence of several concentrations of BAC-C12 and DACMAC-C10. *Bacterial growth in one replicate at 2.5 µg L^−1^.

Growth responses to the exposure of QAACs were different among the tested strains. Most often, lower QAAC concentrations had no effect on the growth kinetics of the strains, while higher concentrations totally inhibited the bacterial growth. For individual strains, inhibitory, but sublethal QAAC concentrations led either to an elongated lag phase without changes of the growth kinetic after the extended lag phase or to an elongated lag phase combined with a changed growth kinetic, often characterized by a reduced doubling time. Growth responses differed among the two different studied QAACs. Strain-specific growth effects are visualized by growth curves in Fig. 1. *E. coli* strain ESBL37B15_13_1E grew in the presence of 5 and 10 µg mL^−1^ of BAC-C12 with the same kinetic as under control conditions without QAAC addition. In the presence of 10 and 15 µg mL^−1^ the same growth kinetics were observed, however, the lag phases were extended by 4 and 12 h, respectively (Fig. 1a). This effect was not observed for DADMAC-C10. The strain showed the same growth kinetic as under control conditions up to the addition of 3.0 µg mL^−1^ of DADMAC-C10 but did not grow at higher concentrations. *E. coli* strain ESBL37B15_13_2E showed a different response kinetic in the presence of BAC-C12. In the presence of 10 and 15 µg mL^−1^ the growth was again as without QAACs. *E. coli* ESBL370B15_13_2A in contrast showed up to concentrations of 10 µg mL^−1^ of BAC-C12 and 2 µg mL^−1^ of DADMAC-C10 the same growth kinetic as under control conditions and did not grow at higher QAAC concentrations. *E. coli* ConF4 showed a slight shift in the lag phase (approximately two hours) in the presence of 5 µg mL^−1^ of BAC-C12 and 1.0 µg mL^−1^ of DADMAC-C10, but a reduced growth rate and a lower final OD at a BAC-C12 concentration of 10 µg mL^−1^ (Fig. 1d).

The studied *Acinetobacter* did not grow in the presence of BAC-C12 and showed the same growth kinetic up to a concentration of 2 µg mL^−1^ DADMAC-C10 as under control conditions and did not grow in the presence of higher DADMAC-C10 concentrations (Fig. 1e).

Plenty of different growth kinetics including reduced growth rates and a reduced final OD (cultured biomass) and extended lag phases were obtained for *P. fluorescens* DSM 50090^T^ after QAAC exposure. This was especially observed in the presence of different BAC-C12 and partially in the presence of different DADMAC-C10 concentrations (Fig. 1f). The *P. fluorescens* strain thereby showed growth in several single wells above the MIC values. This may be due to the growth of a resistant subpopulation present in the inoculum or spontaneously developed within the wells.

*E. faecium* DSM 20477^T^ showed extended lag phases (approx. 4 h) in the presence of 5 and 10 µg mL^−1^ BAC-C12 and 1.5 µg mL^−1^ DADMAC-C10. At lower DADMAC-C10 concentrations, the growth kinetic was identical to that of the control culture (Fig. 1g). *E. faecalis* DSM 20478^T^ showed different growth kinetics in the presence of different BAC-C12 concentrations. In the presence of 10 µg mL^−1^ BAC-C12 the lag phase was shorter but the growth rate higher; in contrast, in the presence of 5 µg mL^−1^ the exponential growth phase started in parallel to the control culture but with a lowered growth rate.

Additionally, Fig. 3 gives a strain-specific and detailed overview of the growth kinetic results for all four strains: *E. coli* ConF4*, E. coli* ESBL37B15_13_1E, *E. faecalis* DSM 20478^T^, and *P. fluorescens* DSM 50090^T^.

### Clay specific effects on the susceptibility of bacteria to QAACs

**Figure 2:**
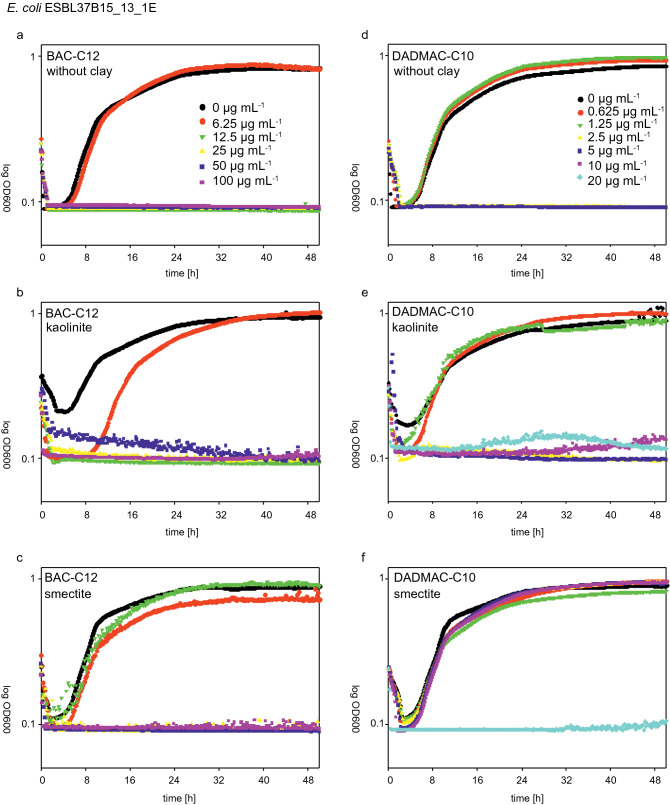
Growth curves of *E. coli* ESBL37B15_13_1E in the presence of different BAC-C12 (**a**–**c**) and DADMAC-C10 (**d**–**f**) concentrations without clay mineral addition (**a**, **d**) and in the presence of smectite (**b**, **e**) and kaolinite (**c**, **f**). Visible are growth curves at QAAC concentrations that were inhibitory in the absence of clay minerals and the presence of kaolinite while growth was not inhibited in the presence of smectite.

The effect of smectite and kaolinite on growth inhibition by QAACs was analyzed with 0.03 mg mL^−1^ of smectite and 0.09 mg mL^−1^ of kaolinite for two *E. coli* strains and the type strains of *P. fluorescens* and *E. faecalis*. For all strains, the addition of smectite apparently increased the MIC values (reduced the QAAC bioavailability) of BAC-C12 by one, partially two concentration steps; for DADMAC-C10 by two to even three concentration steps. In contrast, kaolinite had no effect on the determined MIC values (Table 2). The growth kinetic showed that at strain-specific inhibiting QAAC concentrations, the presence of smectite enabled the bacteria to grow as in the absence of the respective QAACs, as exemplified for strain *E. coli* ESBL37B15_13_1E in Fig. 2. The presence of kaolinite had no effect on the bacterial growth inhibition by either QAAC concentration (Fig. 2a–c). The addition of the two clay minerals itself had no effect on the growth dynamics as was confirmed by the results in control runs without BAC-C12 or DADMAC-C10 addition.

**Table 2:** Shift in apparent MIC [µg mL^−1^] of four tested strains with BAC-C12 and DADMAC-C10 while adding smectite or kaolinite compared to the control with no addition of clay.

Test strain	Clay mineral	MIC	MIC
		BAC-C12	DADMAC-C10
		[µg mL^−1^]	[µg mL^−1^]
*E. coli* ESBL37B15_13_1E	None	12.5	2.5
	Kaolinite	< 6.25/12.5^a^	2.5
	Smectite	25	20
*E. coli* ConF4	None	< 6.25	1.25
	Kaolinite	< 6.25	1.25
	Smectite	12.5/25^a^	10
*P. fluorescens* DSM 50090^T^	None	12.5	2.5
	Kaolinite	12.5	2.5
	Smectite	25	10/20^a^
*E. faecalis* DSM 20478^T^	None	> 6.25	1.25
	Kaolinite	> 6.25	1.25
	Smectite	12.5/25^a^	10

^a^Two values due to differences in duplicates.

**Figure 3:**
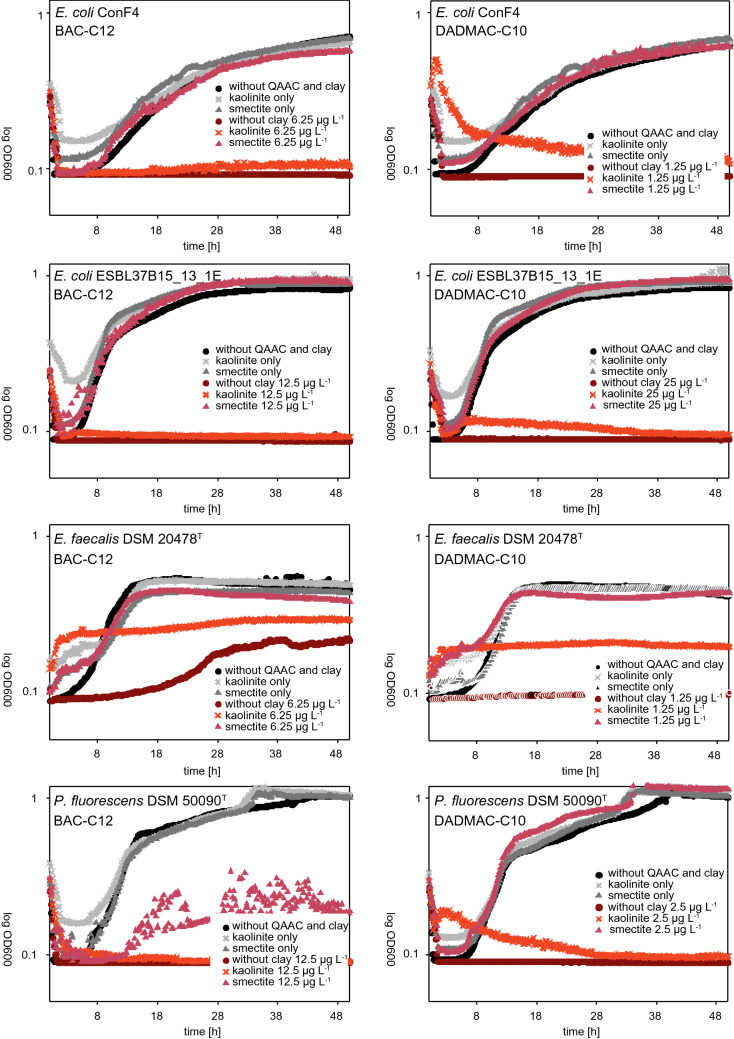
Growth curves of *E. coli* ConF4*, E. coli* ESBL37B15_13_1E, *E. faecalis* DSM 20478^T^, and *P. fluorescens* DSM 50090^T^ in the presence of MICs of BAC-C12 and DADMAC-C10 without clay mineral addition. Same concentrations of QAAC are plotted for kaolinite and smectite experiments. Greyscale plots are without the addition of QAAC while reddish plots are with QAAC.

Only a slight increase of the starting OD values was obtained in some of the measurements, presumably due to an increased turbidity with the clay suspension added. The reduction of acute toxicity in the presence of clay minerals at QAAC concentrations close to MICs is summarized for all tested bacterial taxa and QAACs in Fig. 3.

### Dissolved QAAC concentration versus total concentration

**Figure 4:**
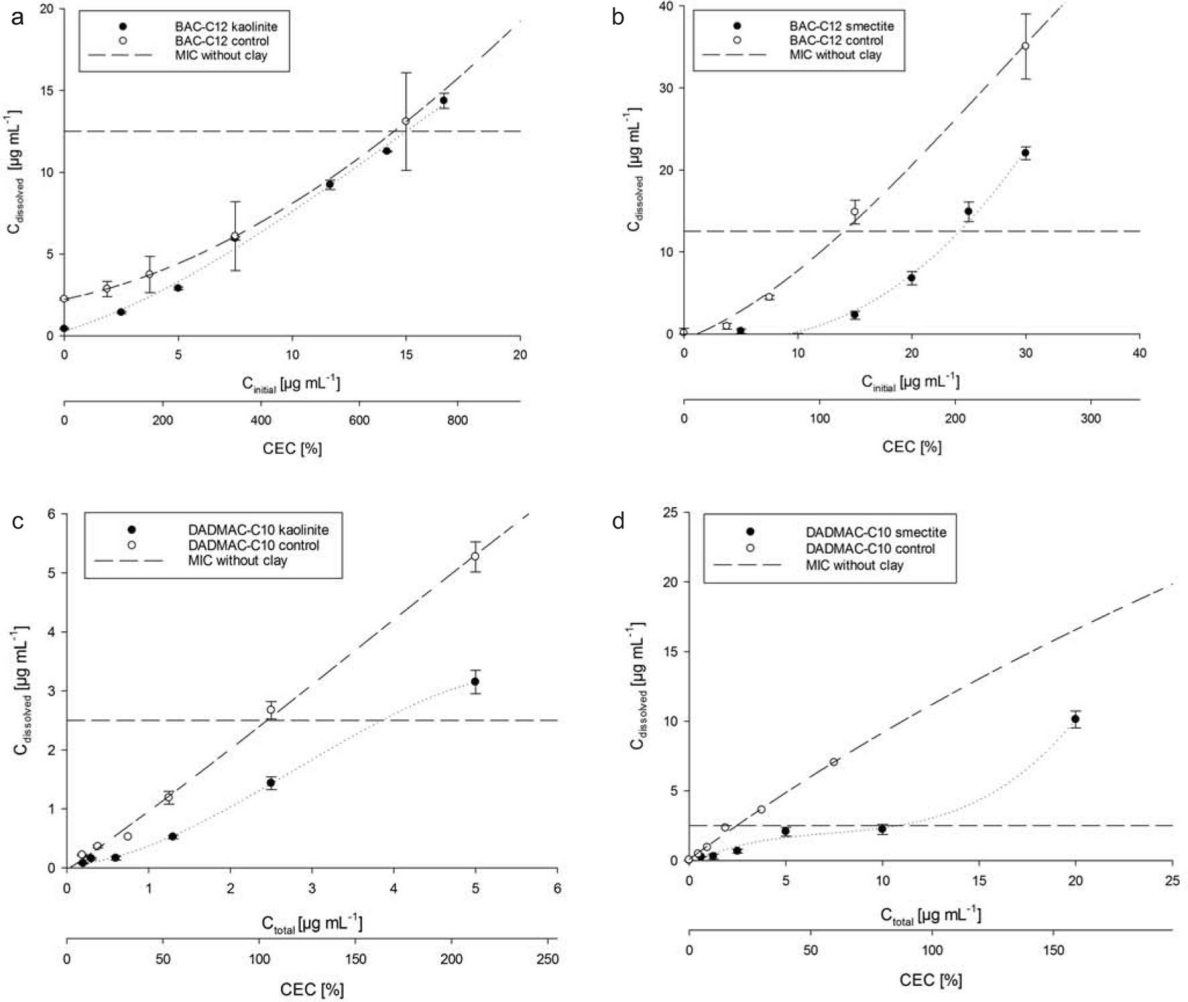
Dissolved concentration [C_dissolved_] versus initial concentration [C_total_] of BAC-C12 and kaolinite (**a**), BAC-C12 and smectite (**b**), DADMAC-C10 and kaolinite (**c**), DADMAC-C10 and smectite (**d**) and MICs shown as long dash lines for *E. coli* ESBL37B15_13_1E and *P. fluorescens* DSM 50090^T^. The according controls without clay minerals are shown as empty circle.

Sorption curves allowed the calculation of dissolved (bio-accessible) QAAC concentrations in the presence of clay minerals from the nominal or total QAAC concentration. This calculation showed that the freely available or dissolved concentration of BAC-C12 hardly changed when kaolinite was added (Fig. 4a). When smectite was added instead of kaolinite, the dissolved concentration of BAC-C12 decreased over the whole tested concentration range compared to the control resulting in a reduction of more than 10 µg mL^−1^ at the nominal concentrations corresponding to the MIC (Fig. 4b).

For DADMAC-C10 concentrations after kaolinite addition, we observed a slight reduction of the dissolved concentration (Fig. 4c), which was, however, smaller than the reduction observed after smectite addition (Fig. 4d). The dissolved concentration of DADMAC-C10 was reduced by about half of the total concentration at concentrations < 5 µg mL^−1^. In contrast, at larger concentrations, the reductions of dissolved concentrations compared to total concentrations were smaller. Addition of smectite was able to reduce the dissolved concentration from 7.5 µg mL^−1^ to 2.5 µg mL^−1^ at total concentration of 10 µg mL^−1^, which is the MIC-value of *E. coli* ESBL37B15_13_1E und *P. fluorescens* DSM 50090^T^ (Fig. 4d). Remarkably, for the curve of DADMAC-C10 with smectite added, the slope of the curve was tentatively s-shaped. The second increase of the slope was observed after the concentration C_dissolved_ corresponding to a saturation exceeding 100% of the CEC of the smectite. This showed that C_dissolved_ stayed below the MIC of 2.5 µg mL^−1^ for total concentrations C_total_ < 10 µg mL^−1^. The data thus demonstrated that sorption occurred beyond the CEC, both in smectite and kaolinite experiments with BAC-C12 and DADMAC-C10. Our data also clearly demonstrated that smectite was able to sequester both, BAC-C12 and DADMAC-C10, so that at nominal MIC the QAACs antimicrobial activity could be buffered by reducing the dissolved concentration of the agents.

Beyond the sorption curves that we obtained, we observed that the addition of QAACs to clay minerals led to flocculation and particle aggregation, as might have been expected from the addition of a salt. This phenomenon was observed especially in smectite samples and became more pronounced at higher QAAC and thus increasing salt concentration.

### TEM analysis and molecular considerations of sorption

**Figure 5:**
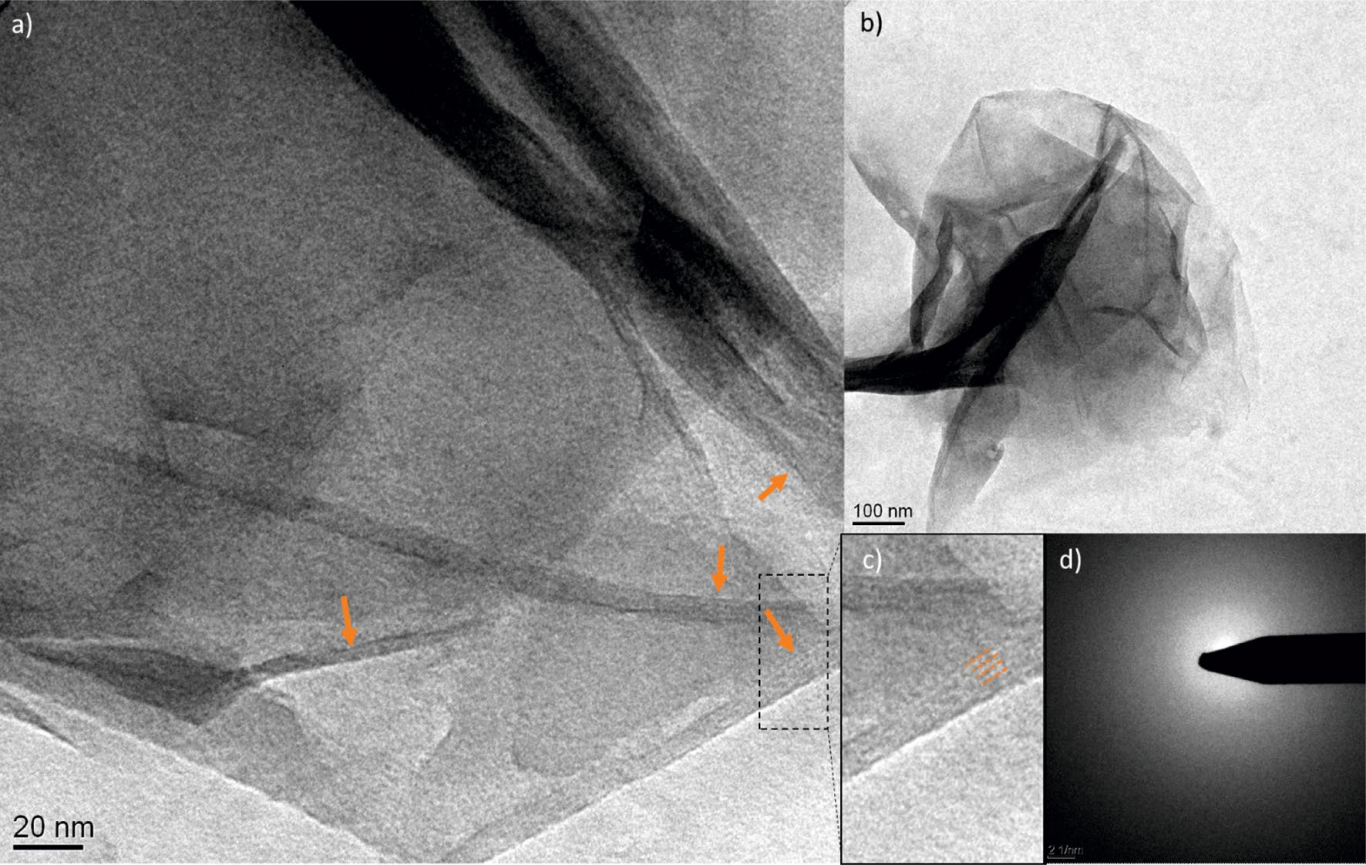
TEM images of a 50 µg BAC-C12 mL^−1^ saturated smectite. Orange arrows (**a**) indicate visible lattice fringes at 81,000 × magnification and crystallographic z-axis. (**b**) Overview of the larger particle or aggregate of particles with typically folded edges. (**c**) Detail demonstrating how three individual layers were identified (orange dashed line); space between lines = 1.99 ± 0.15 nm. (**d**) Diffractogram taken from particle as shown in (**b**) does not show crystallinity due to short-range order of the clay mineral.

Both QAAC-treated and untreated clay mineral samples showed frequent folding or cloud like appearance as is typical for Wyoming bentonite^21^. Diffraction patterns showed diffuse rings or no signs of crystallinities and EDX spectra produced Si/Al-ratios of 1:2 as is typical for smectites. For control and BAC-C12-treated samples, folded edge sites, where lattice fringes of the mineral interlayers were visible, were analyzed by measuring stacks of layers (n = 9) using Image J (W. Rasband, Maryland, USA). Average layer thicknesses were 1.37 ± 0.10 nm for the untreated smectite and 1.99 ± 0.15 nm for the BAC-C12-treated smectite (Fig. 5).

The calculated molar diameters were 0.99 nm and 1.06 nm for BAC-C12 and DADMAC-C10, respectively (Eq. (1), see Table 3). Accordingly, the area occupied by one molecule adsorbed to mineral surfaces equaled roughly 0.77 nm^2^ for BAC-C12 and 0.88 nm^2^ for DADMAC-C10. We used the molar diameter and the BET surface area to estimate the potential number of QAAC molecules adsorbed to the BET surface per ng of smectite and kaolinite. Our estimation did not consider the orientation of the QAACs on clay surfaces. Values for smectite are on the order of 5.5 × 10^10^ molecules DADMAC-C10 per ng and 4.8 × 10^10^ molecules BAC-C12 per ng. Theoretically, one ng of kaolinite provides surface area sufficient for 2.9 × 10^10^ molecules DADMAC-C10 or 2.5 × 10^10^ molecules of BAC-C12. Combining the mass of the QAAC molecule and the potential sorption space of the clays leads to a ratio (ng/ng) of 2.8 × 10^–2^ BAC-C12/smectite (2.6 × 10^–2^ DADMAC-C10/smectite) and a ratio of 1.44 × 10^–2^ for BAC-C12/kaolinite (1.35 × 10^–2^ DADMAC-C10/kaolinite). Considering the experimental conditions, with an addition of 30 µg mL^−1^ smectite and 90 µg mL^−1^ kaolinite, we conclude that theoretically, based on our calculations, the surface was completely covered by QAAC at C_total_ = 1.2 µg mL^−1^ for smectite and at C_total_ = 0.75 µg mL^−1^ of QAAC for the kaolinite experiments.

**Table 3:** Properties of the two QAAC-molecules used in the experiments.

IUPAC name	*N*-Decyl-*N*,*N*-dimethyl-1-decanaminium chloride	*N*-Benzyl-*N*,*N*-dimethyl-1-dodecanaminium chloride
Molecular structure (schematically)		
CAS #	7173-51-5	139-07-1
Chemical formula	C_22_H_48_NCl	C_21_H_38_NCl
Acronym	DADMAC-C10	BAC-C12
CMC [mM]	1.78 ± 0.05	8.3 ± 0.1
Mol. Mass (g/mol)	362	339
mp [°C]	88	42
*d*_m_ [nm]	0.99	1.06

Both belong to the linear alkylammonium compounds. The equivalent molar diameter is given as d_m_. The critical micelle concentration (CMCs) were determined in deionized water according to^50^ using spectrofluormetry and pyren as fluorescens probe.

## Discussion

The performed high throughput growth kinetic measurements of selective Gram-negative and Gram-positive bacterial strains in the presence of different concentrations of two different QAACs combined with two different clay minerals provided information on bacterial strain specific responses to QAACs and the effects of different soil minerals on the QAAC toxicity.

The two different QAACs showed substance specific differences in toxicity. MIC values of DADMAC-C10 were approximately ten-fold lower, indicating a higher toxicity for bacterial growth than BAC-C12^22,23^.

The detailed study of growth kinetics further illustrated the different response of bacterial taxa and even different bacterial strains within one genus to the exposure of QAACs which are often overseen in endpoint MIC measurements (see^24^). The kinetic data showed a broad range of different reactions to QAAC exposure while endpoint MIC determinations differentiated only between endpoint growth as in controls, reduced growth (reduced endpoint OD) or total growth inhibition by specific QAAC concentrations. In contrast, kinetic data also showed that QAAC concentrations, which had no effect on growth measured at the end of the incubation experiment, partially affected bacterial growth by an extension of lag phases or changing growth rates. The growth experiments performed showed for example that different *E. coli* strains exhibit one or both of the aforementioned growth characteristics depending on the QAAC (Fig. 1). Especially the studied *P. fluorescens* strain showed a broad range of different reactions to QAAC exposure, which differed between the two QAACs, different QAAC concentrations, but also among replicate cultures. The growth response of *P. fluorescens* DSM 50090^T^ indicated a high potential for a spontaneous adaptation to QAAC exposure. This was also reported in a previous experiment in which the growth response of the *P. fluorescens* type strain to QAACs was investigated^25^. Changes in the fatty acid composition of the cell membranes or expression of resistance genes as QAAC-efflux were reported^26,27^.

Reported concentrations of QAACs in wastewater range from 9 to 42 µg mL^−1^^28^. Since the MICs determined in our experiments were in a comparable concentration range, an inhibition of the growth of susceptible bacteria seems principally possible. It is important to note in this context that for the selection of resistant strains of bacteria, minimum selective concentrations (MSCs) are more relevant than MICs and MSCs are often several orders of magnitude smaller than MICs^29^, especially when bacteria are exposed to mixtures of chemicals^30^. Hence, a selection of bacteria resistant to QAACs and a potential co-selection of antibiotic-resistant bacteria in wastewater or manure containing QAACs as well as in soils receiving them as fertilizer appears even more probable. However, wastewater, manure, and soils contain particles including clay particles, which can bind QAACs to their surfaces, inactivating them with regard to their effects on bacteria.

For all tested strains, the addition of smectite showed a clear buffering effect on the toxicities of the employed QAACs confirming our hypothesis that 2:1 layer silicates with their interlayer regions, high specific surface areas and high CEC are more effective in reducing the QAAC toxicities than 1:1 layer silicates, which confirms the postulated hypothesis by Mulder et al. (2018)^7^ of interlayer sorption by smectites in environmental systems. Structurally, when compared to kaolinite, interlayer regions (which are not taken into account by the specific surface area) of smectites are of primary interest^31^. When QAACs enter interlayer galleries they can be sequestered from the surrounding medium, so that bacteria are sterically protected from a direct exposure or in other words, the bio-accessible QAAC concentration appears to be reduced in the presence of smectite.

It is remarkable that smectite increased the observed MIC value of DADMAC-C10 more than the MIC of BAC-C12, so we note a compound-specific interaction with clay and sequestration. In contrast to BAC-C12, DADMAC-C10 has two hydrophobic alkyl chains and thus exhibits stronger hydrophobic interaction compared to BAC-C12 with only one C12 alkyl chain (see also Table 3). Ismail et al.^32^ studied sorption of several QAAC homologues including BAC-C12 to sewage sludge and found that sorption affinity correlated positively to hydrophobicity and negatively to critical micelle concentration. A stronger hydrophobic interaction of alkyl chains with clay mineral surfaces and between two layers of QAACs on the clay mineral surfaces could lead to lower accessibility of QAACs for the bacteria and a higher sorption affinity.

However, in our experiments the correlation of MIC to molecular adsorption to the surface was only moderate (Fig. SI-S4; R^2^ = 0.75). Therefore, additional sorption sites should be considered as the cause of the MIC shifts. This is underlined by estimation of surface area occupancy (0.77 nm^2^ and 0.88 nm^2^) based on molar diameters. Giese and van Oss^33^, who performed calculations by layer charge, found that individual QAACs molecules occupy an area between 0.23 nm^2^ and 1.35 nm^2^. Taking into account the clay concentrations used in the experiments, our calculations indicate that the clay surface was completely covered with QAAC molecules at dissolved QAAC concentrations of 0.77 µg mL^−1^ and 1.30 µg mL^−1^. Since MIC shift occurred above those QAAC concentrations, additional mechanism and sorption spaces, besides a single layer sorption to clay surface, influence the shift of the MIC values.

Besides hydrophobic interactions with surfaces, a second sorption mechanism to the clay surface and interlayers of QAACs, is via cation exchange, which leads to a plateau around the CEC where all cations (mostly Mg^+^ in this case) are replaced and thus saturation is achieved. At higher concentrations, QAAC molecules can be adsorbed in an orthogonal arrangement to the mineral surfaces and they might form bi- and multilayer structures that are connected by hydrophobic interaction^34^.

A closer look at nominal MIC values for the kaolinite-amended trials revealed that values for DADMAC-C10 were enhanced stronger than BAC-C12, which is explained by the higher initial QAAC concentration, thus the weak buffering effect of kaolinite steps out more strongly as the surface sorption is the leading process here.

The concentration of dissolved QAACs strongly increased in smectite experiments at sorbed concentrations exceeding 100% CEC (Fig. 4b, d). This was not observed in the kaolinite experiments. As the largest fraction of the CEC of smectite is localized in its interlayer space, this suggests that sorption in the interlayer space was much stronger compared to the outer surface. The results were in good agreement with our hypothesis that the sorption sites of smectites as provided by the CEC play an important role in QAAC sequestration and buffering.

The data of the sorption curves demonstrated that sorption occurred beyond the CEC, both in smectite and kaolinite experiments with BAC-C12 and DADMAC-C10. This is consistent with results of Kwolek et al.^35^, who investigated the sorption of BAC to sodium smectite minerals, but not 1:1 layer silicates.

QAAC adsorption at concentrations exceeding 100% CEC was possibly caused by hydrophobic interaction of the alkyl chains of multiple QAAC molecules. This interaction takes place on the outer surfaces of kaolinite and smectite, but is stronger on smectite due to its higher charge per formula unit, which is commonly 0.25–0.6 in smectite and 0 in kaolinite^36^. In contrast to ion-ion interaction, hydrophobic interaction is a very weak force^37^ and hence bacteria may be able to access the second layer of QAACs at these spots even though the molecules are adsorbed. Zhu et al.^38^ assumed that an alkyltrimethylammonium compound (ATMAC-C16) does not only form lateral mono- and bilayers, but also paraffin-type layers in the interlayer space of montmorillonite when the amount of QAACs exceeds the CEC. Altkyltrimethylammonium compounds are structurally related to BAC and DADMAC; the central nitrogen is surrounded by one alkylchain and three methyl groups. Thus, very low dissolved BAC-C12 concentrations at sorbed concentrations < 100% CEC, compared to DADMAC-C10 indicate that BAC-C12 can enter interlayers more easily. The two alkyl-chains of DADMAC-C10 might hinder entry into the interlayer space. This could also explain the higher MIC buffering potential of smectite in the BAC-C12 experiment (Fig. 4b) compared to DADMAC-C10 (Fig. 4d). Polubesova et al.^39^ showed that adsorption of two short chain BACs with a methyl and the other with an ethyl group (sort of BAC-C1 and BAC-C2) to montmorillonite and illite is limited to 98% and 108% of CEC, respectively, which indicates that the formation of interlayer bilayers is driven by the length and amount of alkyl chains.

We assume that bacterial cells do not come in contact with BAC-C12 and DADMAC-C10 molecules that are sequestered into the interlayer galleries due to size exclusion of bacterial cells. For reference, the used QAACs are between 0.99 nm and 1.06 nm in diameter while bacterial cell sizes are in the order of 1 and 20 µm. This is supported by the fact that basal spacing of alkylammonium cation enriched smectite is between 1.48 nm and a maximum of 4.03 nm^38^.

The observed aggregate formation in our experiments when clay minerals came in contact with QAAC solutions is in line with the results of Penner and Lagaly^40^, who described this effect for alkyltrimethylammonium compounds and smectite, which are similar to BACs and DADMACs. Flocculation did (of course) not occur when clay was mixed with pure water and it was stronger for smectite suspensions in comparison to kaolinite suspensions. The presumable causes of this effect is on the one hand that QAACs carry a permanent cationic charge at the nitrogen that is attracted to the negatively charged surface of smectite particles. When adsorbed to the clay surfaces the positive charge might be still available for other negatively charged surfaces and thus further clay particles are attracted towards the QAAC molecule, which is holding the aggregates together. On the other hand, the repulsive forces of negatively charged clay surfaces is lowered by the absorbed QAAC. Thus, the attractive forces (e.g. van der Waals forces) prevail over repulsive forces and the net interaction potential leads to flocculation. Derjaguin, Landau, Verwey, and Overbeek described this so-called DLVO theory at first^41^. Especially the QAAC molecules inside those aggregates that are surrounded by smectite minerals might not be accessible for bacteria. This effect is less pronounced in kaolinite experiments as expected, since the charge of kaolinite surfaces is neutral.

The results confirm our hypothesis that 2:1 layer silicates like smectite are more effective in increasing the apparent MIC as non-expandable clay minerals. But we have to acknowledge that not only the CEC, but also the surface area of smectites as well as the charge density is greater than in kaolinites and may influence this MIC value shift.

TEM images document interlayer sorption and expansion of the basal spacings by QAACs to smectite. The imaging data are in good agreement with previous data based on X-ray diffraction by Kwolek et al.^35^ that showed interlayer space of smectites increased twice as much by BACs with alkyl chain lengths greater than ten as with alkyl chain lengths smaller than ten. This provided strong visible evidence for the interlayer sorption of BAC-C12 to smectites as reported more generally for QAACs to 2:1 expandable layer silicates determined by x-ray diffraction^42^.

## Conclusions

We were able to confirm our hypothesis that there is a strain specific growth response to QAACs. This response differs between BAC-C12 and DADMAC-C10 by a factor of ten. Minimum inhibitory concentrations for the test strains were in a concentration range comparable to concentrations reported for wastewater. Considering that minimum selective concentrations are commonly several orders of magnitude smaller than MICs, this suggests that a selection of QAAC resistant bacterial strains could principally occur in wastewater, manure or soils receiving both as fertilizer. Since wastewater, manure and soils contain particles including clay minerals that can sorb QAACs, the magnitude of the effect of sorption on the inhibition of bacterial growth by QAACs is essential for assessing the likelihood of the selection of resistant bacteria. Sorption of QAACs to clay minerals can also explain how sensitive bacteria as the tested manure derived *Acinetobacter* strain can survive in a habitat containing concentrations of QAACs which are in the range of MICs.

Shifts of apparent MICs to larger total QAAC concentrations in combination with sorption curves prove that sorption of QAACs to 2:1 expandable clay minerals reduces their bio-accessibility and acute toxicity. Kaolinite was no efficient adsorbent. The magnitude of the MIC shift depends on the QAAC structure as well as the clay mineral structure. Our calculations confirmed that BET surfaces are not sufficient to explain amounts QAAC adsorbed and that interlayer sorption for smectite likely plays a dominant role.

Independent microscopic observations of increased interlayer distance of QAAC-treated smectite compared to untreated smectite together with the stronger reduction of toxicity in the presence of smectite compared to kaolinite point to the relevance of interlayer sorption of QAACs for the detoxification. In addition, the entrapment of QAACs in flocs and aggregates of clay mineral particles potentially causes a further reduction of bio-accessibility. Future experiments should clarify the effect of natural organic matter and micro-aggregation on the detoxification of an extended spectrum of different QAACs. Further research on the toxicity buffering effects of e.g. soil organic matter and micro aggregate formation is needed. In order to get a toxicity ranking expressed by MIC values, ATMACs should be tested in future works as well as QAACs with different chain length, since BAC-C12 and DADMAC-C10 are representing only two of the three main QAAC groups.

## Materials and methods

### Chemicals and materials

In view of the complexity of the system soil under study, it was our aim to address our central hypothesis by using model compounds. BAC-C12 and DADMAC-C10 were chosen as they belong to the most frequently encountered QAACs in the agricultural environment^2^. BAC-C12 consists of a benzyl, two methyl and one dodecyl group. DADMAC-C10 is composed of two methyl and two decyl groups. Both have a positively charged nitrogen cation, thus both are organic salts. Table 3 gives an overview of the structure and the main properties of these two QAACs.

High purity water (MQ) was prepared by a Milli-Q water purification system (Millipore) and used for all mineral and QAAC stock and working solutions, dilutions and HPLC (high pressure liquid chromatography)-solvent preparation. Samples and solutions were centrifuged either with a Micro Star 17R centrifuge (VWR) when the volume was below 2 mL or Rotanta 460R (Hettich) for volumes > 2 mL. BAC-C12 (> 98.0%) was obtained from TCI (Tokyo, Japan) and DADMAC-C10 (≥ 99.0%) purchased from Glentham Life Science (Wiltshire, UK).

The clay minerals used in the studies were the 2:1—layer silicate montmorillonite MX-80 as smectite (AMCOL, Cheshire, England) and the 1:1—layer silicate kaolinite pharmakaolin (ZIEGLER Minerals, Wunsiedel, Germany). The cation exchange capacity (CEC) was 106.54 cmol_c_ kg^−1^ and 6.65 cmol_c_ kg^−1^ for smectite and kaolinite, respectively. The Brunauer–Emmett–Teller specific surface area (BET) as determined via N_2_-physisorption-isotherm was 42 m^2^ g^−1^ and 22 m^2^ g^−1^ for smectite and kaolinite, respectively (Table 4, with schematic mineral structures).

**Table 4:** Properties of the clay minerals used in the experiments.

Name	Montmorillonite	Kaolinite	
Molecular structure (schematically)			
Brand	Volclay	Pharmakaolin B860	
Supplier	AMCOL Specialty Minerals (Cheshire, England)	ZIEGLER Mineralstoffe (Wunsiedel, Deutschland)	
Chemical formula	(Na,Ca)_0.33_ (Al_1.67_Mg0.33) Si_4_O_10_(OH)_2_ · nH_2_O ^a^	Al_2_Si_2_O_5_(OH)_4_^b^	
BET surface area [m^2^ g^−1^]	42	22	
CEC [cmol_c_ kg^−1^]	106.5	6.7	

The cation exchange capacity (CEC) of the minerals was determined with the Cu-triethylenetetramine-complex-method. Specific surface areas were derived from BET sorption isotherms.

^a^^51^.

^b^^52^.

For the clay purification process, a 1 N sodium acetate buffer (H_3_CCOONa, ≥ 99.5%, Merck; CH_3_COOH 100%, Merck), sodium carbonate solution 0.125 g L^−1^ (Na_2_CO_3_, 99.9%, Merck), H_2_O_2_ (30%, Merck) and 1 M NaOH (Titrisol, Merck) were used. For the CEC determination, triethylenetetramine (≥ 97%, Sigma-Aldrich) and dehydrated copper(II) sulfate pentahydrate (Ph Eur, Merck) were used in order to prepare a 0.01 mol L^−1^ Cu-triethylenetetramine color complex solution.

Acetonitrile (100%, HiPerSolv Chromanorm, VWR), formic acid (≥ 98%, Rotipuran, Carl Roth) and ammonium formate (99%, Acros Organics) were used to prepare HPLC eluents as well as all QAAC stock and working solutions.

### Bacterial strains

Eight bacterial strains (Table 5) were selected for the experiments, which represent taxa of pathogenic bacteria causing immense healthcare problems especially due to the development of antibiotic resistances. Those taxa are present in manure and wastewater and represent nosocomial pathogens which can survive in nature (soil) after their release.

**Table 5:** Overview of bacterial strains applied in this study.

Species	Strain	Isolation source	References
*Escherichia coli*	ESBL37B15_13_1E	Manure	*Schauss *et al*.* (2015)
*Escherichia coli*	ESBL232B15_13_2E	Manure	*Schauss *et al. (2015)
*Escherichia coli*	ESBL370B15_13_2A	Biogas plant digestate	*Schauss *et al*.* (2015)
*Escherichia coli*	ConF4	K12 variant	
*Acinetobacter* sp.	KPC-SM-21	Biogas plant digestate	Mishra (2014)
*Pseudomonas fluorescens*	DSM 50090^T^	Biofilter	Migula (1900)
*Enterococcus faecium*	DSM 20477^T^	–	Schleifer & Kilpper-Bälz (1984)
*Enterococcus faecalis*	DSM 20478^T^	–	Schleifer & Kilpper-Bälz (1984)

–, Source not known.

Bacterial growth experiments were performed with four *E. coli* strains, the type strain of *P. fluorescens,* an *Acinetobacter* strain (all *Gammaproteobacteria,* Gram-negative bacteria), and the type strains of *E. faecalis* and *E. faecium* (Firmicutes, Gram-positive bacteria) (Table 5). Strains were isolated either from environmental samples in previous studies or obtained from type culture collections (Table 5). Strains were selected because genome sequences were available for all of them and they mostly originate from manure and soil. All strains were assigned to the species level and well characterized with respect to their antibiotic resistances and physiological properties. All strains were pre-cultured on Mueller–Hinton agar (MHA, Carl Roth) at 37 °C for 24 h. For long-term preservation fresh bacterial biomass was suspended in Gibro newborn calf serum (NBCS, ThermoFisher Scientific) and stored at − 20 °C.

### Bacterial growth experiments and MIC value determination

Bacterial growth response to QAACs (BAC-C12 and DADMAC-C10) exposure and the determination of QAAC specific MIC values were performed in microdilution assays according to the standard procedure for antibiotic susceptibility testing given by the Clinical Laboratory Standards Institute CLSI^43^ guidelines. All experiments were performed in Mueller–Hinton broth (MHB) in a total volume of 200 µl using transparent flat 96 well microtiter plates (Greiner Bio-One GmbH) covered with sterile transparent plastic lids. Each well was preloaded with 100 µL MHB (control wells without QAACs) or 50 µL double concentrated MHB mixed with 50 µL four-fold concentrated QAAC solutions. The used QAAC stock solutions were dissolved in autoclaved pure water and filtered using 0.45 µm sterile cellulose-acetate filter units (VWR). The stock solution was stored at 4 °C and refreshed monthly. Eleven different QAAC concentrations were tested in parallel with a growth control without QAAC addition. The final QAAC concentrations were in the range of 5 to 55 µg mL^−1^ for BAC-C12 and 0.5–5.5 µg mL^−1^ for DADMAC-C10 (with a gradual increase by 5 µg mL^−1^)*.* The applied concentration ranges for BAC-C12 and DADMAC-C10 were based on the range of the environmental BAC-C12 concentration (9–42 µg mL^−1^) in wastewater since data for soils are currently not available^28^. In preliminary tests with a larger range of QAAC concentrations (data not shown) we found that the tested strains all had inhibited growth in concentrations ranges between 5 and 30 µg mL^−1^. Further evaluation in preliminary tests had shown that DADMAC-C10 was ten times more toxic for bacteria as BAC-C12. Thus, DADMAC-C10 concentrations were selected accordingly. Immediately before the incubation experiments were started, 100 µL inoculated MHB was added to each well. The MHB cell suspensions were generated as follows: an inoculation loop full of fresh overnight cultured bacterial biomass was suspended in 6 mL of an autoclaved 0.9% (w/v) NaCl solution to a McFarland standard density of 0.5 which provides an optical density comparable to the density of a bacterial suspension of 1.5 × 10^8^ colony forming units (CFU) mL^−1^. For Gram-negative bacteria 109 µL and for Gram-positive bacteria 218 µL of the suspensions were used for the inoculation of 12 mL MHB. Microtiter plates were incubated for 48 h at 25 °C in an Infinite M200 or Infinite F200 spectrophotometer (Tecan; Germany). The bacterial growth was monitored during incubation by a continuous optical density (OD) measurement at a wavelength of 600 nm in 10 min intervals. Before each OD measurement the microtiter plates were shaken for 15 s with an amplitude set to 6. Growth curves were displayed in SigmaPlot 13.0 (Systat). The lowest concentration above the last concentration of the QAACs that showed bacterial growth represents the MIC.

### Growth experiments elucidating the buffering effect of clay minerals on QAAC toxicity

The potential buffering effect of clay minerals on the susceptibility of bacterial cultures to the exposure of QAACs (MIC value shift) was tested as described above in 96-well plate test systems. Concentrated QAACs and clay mineral solutions were therefore pre-mixed and equilibrated for 30 min in 2 mL Eppendorf tubes before they were added to microtiter plates and inoculated with bacterial biomass. The nominal QAAC concentrations (uncorrected for sorption), that were chosen around the predetermined MIC values, were 6.25, 12.5, 25, 50, and 100 µg mL^−1^ for BAC-C12 and 0, 0.625, 1.125, 2.5, 5.0, 10, and 20 µg mL^−1^ for DADMAC-C10, respectively.

In a preliminary trial different clay mineral concentrations were tested in parallel. Subsequently, the clay mineral concentrations for the growth experiments were chosen in a way that the highest concentration of QAAC employed corresponded to the CEC value of 76.4 cmol_c_ kg^−1^ for smectite^44^. As kaolinite is not capable of interlayer sorption, the amount of kaolinite in the incubation experiments were adjusted in order to provide identical BET surface areas as present in the smectite experiments. The potential interlayer surfaces that can hypothetically be accessed by QAACs are only present in smectites and are not expressed by the BET surface area values of purified clays given in Table 4. The larger specific surface area for smectite compared to kaolinite is a result of their smaller crystallite size.

First tests included final smectite and kaolinite concentrations in the range of 0.016 to 0.064 mg mL^−1^ and 0.094 to 0.374 mg mL^−1^, respectively. Finally, clay mineral concentrations of 0.03 mg mL^−1^ (smectite) and 0.09 mg mL^−1^ (kaolinite) were selected for the further experiments. Growth controls included bacterial growth in MHB without any additives and in the presence of clay minerals. Each control and each QAAC/clay ratio was tested in duplicate (Tab. SI-S1-S4).

### Clay preparation and characterization

Before kaolinite and smectite were used in the experiments, a pretreatment was carried out in order to purify them; the associated components carbonate and organic matter were removed and a subsequent particle size fractionation allowed for a homogenous clay fraction (≤ 2 µm). The method applied was adapted from Tributh and Lagaly^45^. For carbonate removal, a 1 N sodium acetate buffer solution (H_3_CCOONa, CH_3_COOH) adjusted to pH = 5 was added to each clay type. The suspensions were stirred periodically while heating to 90 °C in a water bath until bubbling ceased. Suspensions were then centrifuged at 920 g for 30 min and the clear supernatant was discarded. This process was repeated twice, omitting the heating.

Organic matter and sulfide traces were removed by subsequently adding H_2_O_2_ in a pH = 5 sodium acetate buffer. The sample was then re-suspended and the suspension heated to 65 °C and H_2_O_2_ added every hour until bubbling ceased. In order to decompose the remaining H_2_O_2_, the suspension was brought to a slight boil just below 100 °C. The supernatant was poured off after centrifuging at 920 g for 30 min.

All clay samples were dispersed with pH = 10 sodium carbonate solution. The pH values were checked and if necessary adjusted to 8.5–9.5 with NaOH prior to particle size fractionation. Separation of particles < 2 µm was accomplished by centrifugation (Rotanta centrifuge, Hettich, Tuttlingen, Germany) for 6 min at 100 g according to Stokes law. The supernatant containing the < 2 µm fraction was decanted and collected. The sample dispersion and centrifugation step was repeated three times. To reduce the volume of the gained clay suspension, NaCl was added to flocculate the sample. After decanting the clear supernatant, the remaining suspension was washed with deionized water (DI water) until the electric conductivity was smaller than 20 µS cm^−1^. The exact clay concentration of the suspension was determined gravimetrically by drying suspension aliquots at 105 °C.

Determination of the CEC was performed with copper triethylenetetramine [Cu(trien)]^2+^ complex using the method of Meier and Kahr^46^ as modified by Ammann^47^. The [Cu(trien)]^2+^color complex solution was added to 100 mg of lyophilized smectite or kaolinite and the mixture was shaken for 30 min. The samples were centrifuged at 2950 g for 30 min. Three mL of the supernatant were transferred into cuvettes and its extinction measured with a photometer (T80 UV/Vis Spectrometer, PG Instruments Ltd) at 577 nm. The CEC was calculated from the concentrations of the [Cu(trien)]^2+^ color complex according to Ammann^47^. Measurements were performed in triplicate.

The surface area of porous solids and fine powders such as dried clay minerals can be measured by gas adsorption^48^. The BET method was used to determine the specific surface area of the clay minerals. To this end, 100 mg of freeze-dried clay from the pretreated smectite and kaolinite suspensions were heated to 120 °C for 16 h in order to desorb air moisture. The adsorption and desorption of N_2_ was measured with a Quadrasorb evo (Quantachrome, Boynton Beach, USA) and the BET sorption isotherm served to determine the specific surface area^48^.

Transmission electron microscopy (CM30 Phillips TEM with EDAX 9900 EDX-Detektor, 300 kV) was used in order to observe fine structural changes of smectite particles upon QAAC treatment. Smectite suspensions containing 0.3 mg smectite mL^−1^ were mixed with 0, 30, 50, and 2000 µg mL^−1^ of BAC-C12 in polypropylene centrifuge tubes and allowed to equilibrate at 250 rpm on an orbital shaker for 1 h. Smectite samples were mounted onto a carbon-coated copper grid (Plano, Wetzlar, Germany) from suspension. For each sample we intended to investigate a minimum of four locations on the grid where we could record images in a magnification range of 10,500× to 110,000×. Control and 50 µg mL^−1^ BAC-C12 treated smectite grids were used for systematic analysis.

### Characterization of QAACs

We used the equivalent molar diameter *d*_m_ to get an estimate of the size of QAAC molecules. This simplified model assumes a spherical structure but is considered to be adequate in order to get an idea of the molecule size^49^.

Based on the molar volume $$\left( {V_{m} = M* \rho^{ - 1} } \right)$$$$d_{m}$$ is calculated as follows:

1 $$d_{m} = 2*\left( {\frac{{3*V_{m} }}{4*\pi *N}} \right)^{\frac{1}{3}}$$

with *N* equal to Avogadro's number, *ρ* the compounds’ density (g m^−1^) and the molar mass *M* (g mol^−1^). As an approximation, the area occupied by QAAC molecules is calculated with the following equation:

2 $$A_{m} = \frac{{\pi * d_{m}^{2} }}{4}$$

Additionally the absolute mass of the QAAC molecules was determined by dividing the molar mass by Avogadro's number. The maximum loading of the clay surface area is assumed by taking account of the absolute mass and the molecule size of the QAAC, as well as the BET surface area of clays.

The critical micelle concentration (CMC) is a parameter that is typically reported for surfactants and denotes the concentration, above which a compound self-assembles to form micelles with the polar (in our case cationic) head groups pointing outwards in a polar solvent. Below the CMC, QAACs occur dissolved as single molecules, similar to other electrolytes, with a tendency to accumulate at surfaces and phase boundaries. Above the CMC, the micelles formed rather act as a second phase, comparable to hydrocarbon droplets, that could for example solubilize other hydrophobic compounds in the system. For our study, the CMCs of BAC-C12 and DADMAC-C10 were determined using spectrofluorometry and pyrene as sensor to the different solubilization caused by micellization. The peak intensity ratio was used to derive the CMC values according to Aguiar et al.^50^.

### BAC-C12 and DADMAC-C10 sorption curves

In order to assess the dissolved concentration (C_dissolved_) of BAC-C12 or DADMAC-C10 in equilibrium with clay minerals, the conditions of the experimental design in the MIC-incubation-experiments (section “TEM analysis and molecular considerations of sorption”) were mimicked at a larger scale for sorption curves, thereby providing enough sample volume for the required HPLC-tandem mass spectrometry (MS/MS) measurement and analysis. QAAC concentration ranges were chosen to encompass the respective MIC values. Compared to the MIC-experiments, smaller concentration range steps (for a finer resolution) were selected. Concentrations for smectite (0.03 mg mL^−1^) and kaolinite (0.09 mg mL^−1^) added remained constant. Smectite or kaolinite were equilibrated with different concentrations of QAACs in 20 mL amber glass vials shaken overhead for 30 min, analogous to the QAAC and clay mixing in 2 mL Eppendorf tubes. Two mL of this solution were transferred again to 20 mL empty amber glass vials and mixed with 2 mL of MHB (Sigma Aldrich) (44 g L^−1^) and 8 mL of bacterial blank solution, which is equivalent to the conditions in the well plates described in section “TEM analysis and molecular considerations of sorption”. After shaking with a vortex shaker (VORTEX 3, IKA, Germany) and centrifuging at 710 g for 30 min, aliquots of 30–120 µL of the clear supernatants were transferred to 2 mL clear glass vials and made up to 1 mL total volume with acetonitrile for the HPLC–MS/MS measurement. The experiments were performed in triplicate. For each experiment, a seven point calibration in acetonitrile was prepared.

For separation of QAACs from other organic compounds, a Waters 2690 Separations Module, equipped with a Waters XSelect CSH Phenyl-Hexyl column (3.5 µm particle size, 2.1 mm inner diameter × 150 mm length) and a guard XSelect column of the same material (3.5 µm particle size, 2.1 mm inner diameter × 5 mm length) was used. Column temperature was set at 37 °C and an isocratic flow of 0.3 mL min^−1^ with 15% solvent A (MQ-water and 50 mM formic acid and 10 mM ammonium formate) and 85% solvent B (acetonitrile) and 20 µL injection volume were chosen. The matrix effects of MHB on separation and detection were tested and could be neglected at the applied concentrations of maximally 3.66% (v/v). A Micromass Quattro Micro triple quadrupole mass spectrometer operating in positive ion multiple reaction-monitoring mode was used for mass detection (further settings in SI-S7). Peak integration was performed with MassLynx Quanlynx (Waters), data analysis with Microsoft Excel 2010 and curve fitting with SigmaPlot 12 (Systat).

## Supplementary information

Supplementary Information.
