# Supplementary material for: Smectite clay minerals reduce the acute toxicity of quaternary alkylammonium compounds towards potentially pathogenic bacterial taxa present in manure and soil
Source: Sci Rep. 2020 Sep 21;10:15397. doi: 10.1038/s41598-020-71720-5 (PMC7505985; doi:10.1038/s41598-020-71720-5)
Supplement: Supplementary file 1 — Supplementary Information. [file 41598_2020_71720_MOESM1_ESM.pdf]

Supplementary data

Smectite clay minerals reduce the acute toxicity of quaternary alkylammonium compounds towards potentially pathogenic bacterial taxa present in manure and soil

Benjamin Justus Heyde[1#], Stefanie P. Glaeser[2*,#], Linda Bisping[1,2], Kristin Kirchberg[3], Rüdiger Ellinghaus[3], Jan Siemens[1], Ines Mulder[1*]

[1] Institute of Soil Sciences and Soil Conservation, iFZ Research Centre for Biosystems, Land Use and Nutrition, Justus Liebig University, Heinrich-Buff-Ring 26-32, 35392 Gießen

[2] Institute for Applied Microbiology, iFZ Research Centre for Biosystems, Land Use and Nutrition, Justus Liebig University, Heinrich-Buff-Ring 26-32, 35392 Gießen

[3] Institute of Physical Chemistry, Justus Liebig University, Heinrich-Buff-Ring 17, 35392 Gießen

[#]shared first authors

[*]corresponding authors

Corresponding authors:

Dr. Stefanie Glaeser (microbiology)

Stefanie.Glaeser@umwelt.uni-giessen.de

Dr. Ines Mulder (soil science)

Ines.Mulder@umwelt.uni-giessen.de

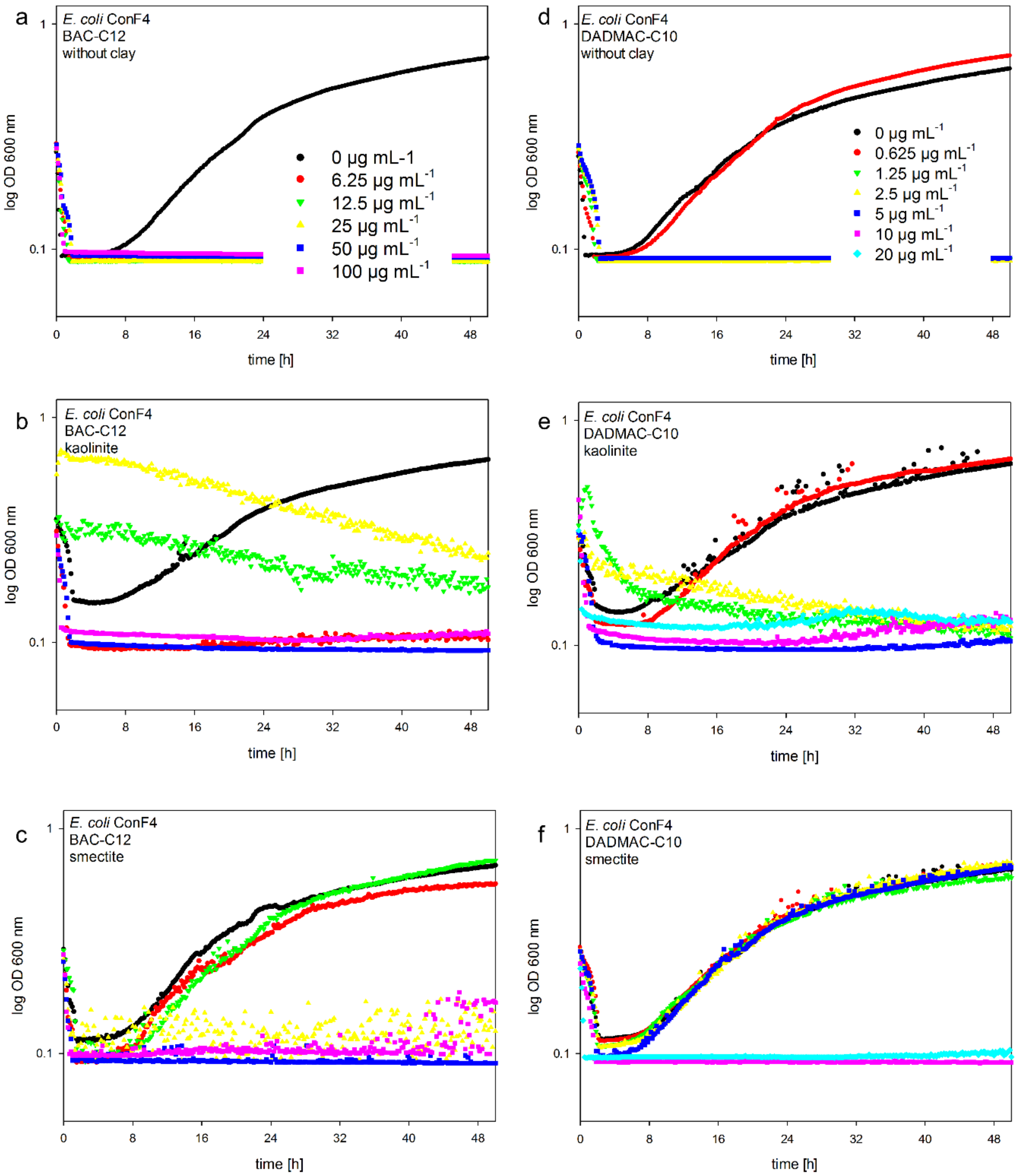

Figure S1 Growth curves of strain *E. coli* ConF4 in the presence of different BAC-C12 (a-c) and DADMAC-C10 (d-f) concentrations without clay mineral addition (a, d) and in the presence of kaolinite (b, e) and smectite (c, f). QAAC concentrations marked with dashed lines represent concentrations which were inhibitory in the absence of clay minerals and the presence of kaolinite while growth was non-inhibited in the presence of smectite.

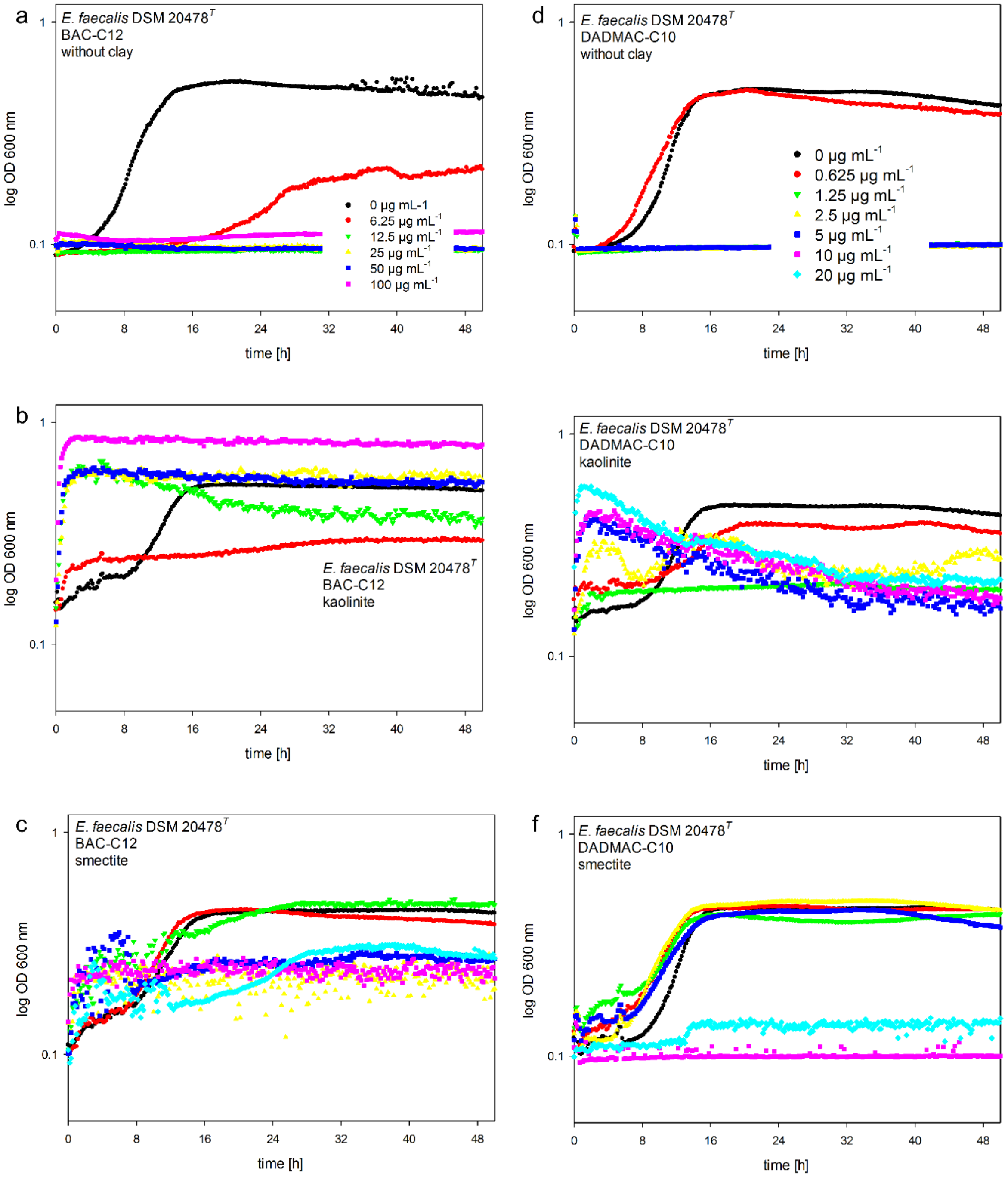

Figure S2 Growth curves of strain *E. faecalis* DSM 20478^T in the presence of different BAC-C12 (a-c) and DADMAC-C10 (d-f) concentrations without ton mineral addition (a, d) and in the presence of smectite (b, e) and kaolinite (c, f). QAAC concentrations marked with dashed lines represent concentrations which were inhibitory in the absence of ton minerals and the presence of kaolinite while growth was non-inhibited in the presence of smectite.

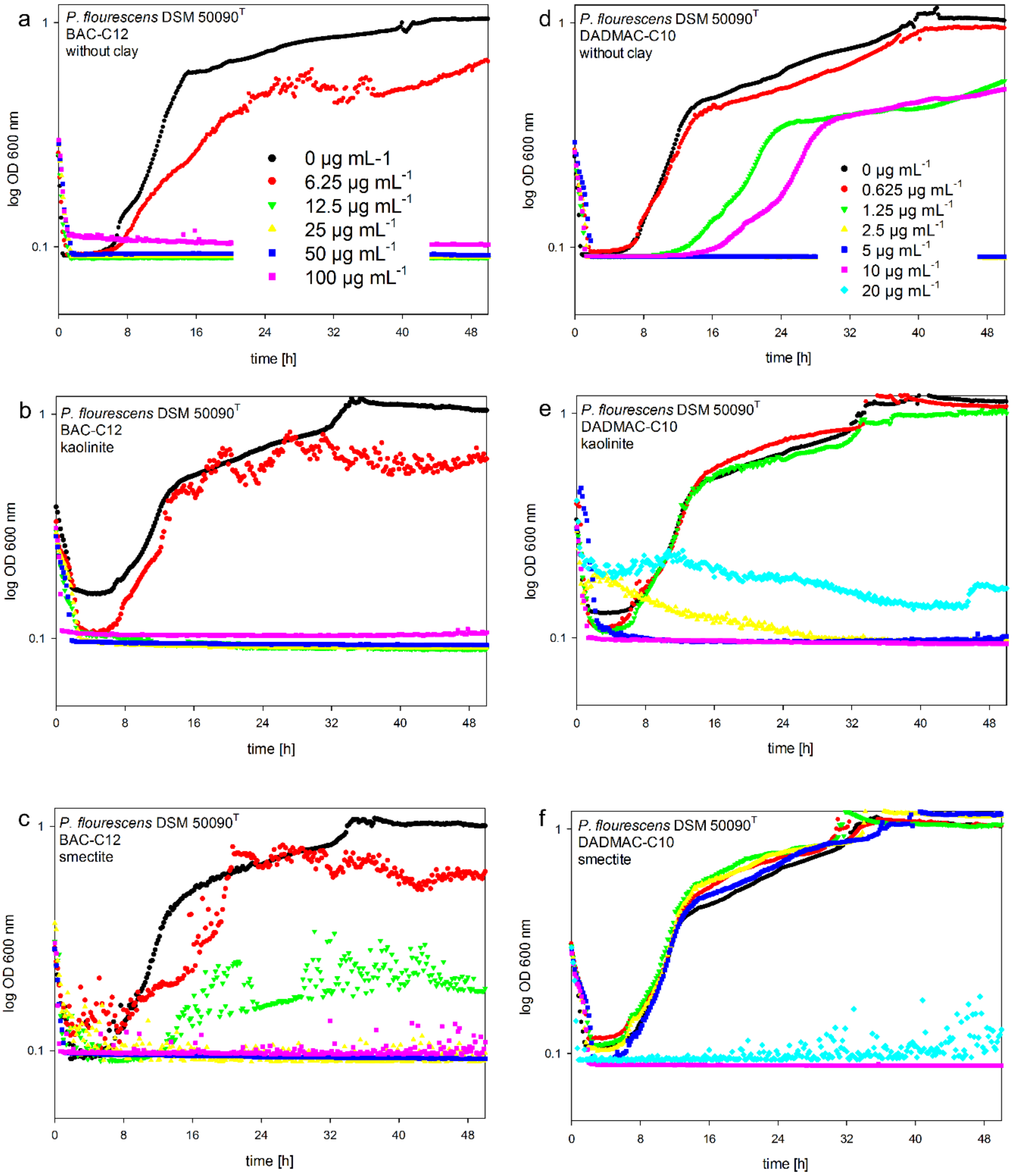

Figure S3 Growth curves of strain *P. fluorescens* DSM 50090^T in the presence of different BAC-C12 (a-c) and DADMAC-C10 (d-f) concentrations without ton mineral addition (a, d) and in the presence of smectite (b, e) and smectite (c, f). QAAC concentrations marked with dashed lines represent concentrations which were inhibitory in the absence of ton minerals and the presence of kaolinite while growth was non-inhibited in the presence of smectite.

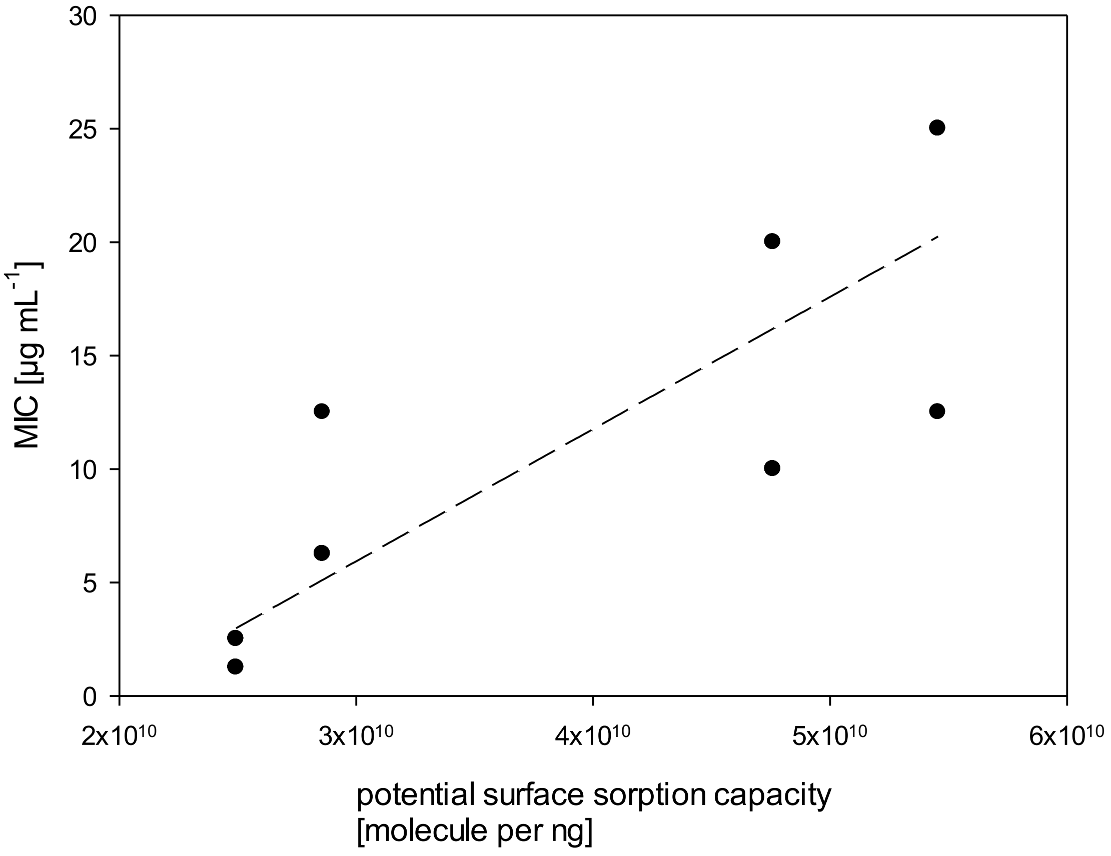

Figure S4 Correlation of all MIC values vs potential surface sorption capacity of BAC-C12 or DADMAC-C10 on kaolinite or smectite surface ($R^2 = 0.75$). Four treatments are BAC-C12 / kaolinite, BAC-C12 / smectite, DADMAC-C10 / kaolinite and DADMAC-C10 smectite. MIC values of *E. coli* ESBL 37 B15_13_1E, *E. coli* ConF4, *P. fluorescens* DSM 50090^T and *E. faecalis* DSM 20478^T are shown and some overlap others

Table S1 Schematic structure of the 96-well plates as used in the final tests with kaolinite and BAC-C12 0 – 100 μg mL^{-1}

	Without clay						Kaolinite [93.5 μg mL^{-1}]					
BAC-C12 [μg mL^{-1}]	0	6.25	12.5	25	50	100	0	6.25	12.5	25	50	100
E. coli	+	+					+	+				
ESBL37B15_	+	+					+	+				
13_1E												
P. fluorescens	+	+					+	+				
DSM 50090^{T}	+	+					+	+				
E. coli ConF4	+						+					
	+						+					
Without bacteria												

Table S2 Schematic structure of the 96- well plates as used in the final tests with smectite and BAC-C12 [0 – 100 μg mL^{-1}]

	Without clay						Smectite [32.17 μg mL^{-1}]					
BAC-C12 [μg mL^{-1}]	0	6.25	12.5	25	50	100	0	6.25	12.5	25	50	100
E. coli	+	+					+	+	+			
ESBL37B15_	+	+					+	+	+			
13_1E												
P. fluorescens	+	+					+	+	+			
DSM 50090^{T}	+	+					+	+	+			
E. coli ConF4	+						+	+	+			
	+						+	+	+			
Without bacteria												

Table S3 Schematic structure of the 96- well plates as used in the final tests with kaolinite and DADMAC-C10 [0 – 20 μg mL^{-1}]

	Without clay					Kaolinite [93.5 μg mL^{-1}]						
DADMAC-C10 [μg mL^{-1}]	0	0.625	1.25	2.5	5.0	0	0.625	1.250	2.5	5	10	20
E. coli	+	+	+			+	+	+				
ESBL37B15_	+	+	+			+	+	+				
13_1E												
P. fluorescens	+	+	+			+	+	+				
DSM 50090^{T}	+	+	+			+	+	+				
E. coli ConF4	+	+	+			+	+	+				
	+	+				+	+	+				
Without bacteria												

Table S4 Schematic structure of the 96- well plates as used in the final tests with smectite and DADMAC-C10 [0 – 20 µg mL^{-1}]

	Without clay					Kaolinite [93.5 µg mL^{-1}]						
DADMAC-C10 [µg mL^{-1}]	0	0.625	1.25	2.5	5.0	0	0.625	1.250	2.5	5	10	20
E. coli ESBL37B15_13_1E	+<br>+	+<br>+	+<br>+			+<br>+	+<br>+	+<br>+	+<br>+	+<br>+	+<br>+	
P. fluorescens DSM 50090^T	+<br>+	+<br>+	+<br>+			+<br>+	+<br>+	+<br>+	+<br>+	+<br>+	+<br>+	
E. coli ConF4	+<br>+	+<br>+				+<br>+	+<br>+	+<br>+	+<br>+	+<br>+		
Without bacteria												

Table S5 Schematic structure of the 96-Well Plates as used in the sensitivity tests of MO (*E. coli* ESBL37B15_13_1E; *E. coli* ESBL232B15_13_2E; *E. coli* ESBL370B15_13_2A; *E. coli* ConF4; *E. faecium* DSM 20477^T; *E. faecium* DSM 20478$^{T;}$ *Acinetobacter* sp. KPC-SM-21; *P. fluorescens* DSM 50090^T; *P. fluorescens* DSM 50090^T BAC-C12 (F6); *P. fluorescens* DSM 50090^T BAC-C12 (E11)) to QAAC (BAC-C12 and DADMAC-C10)

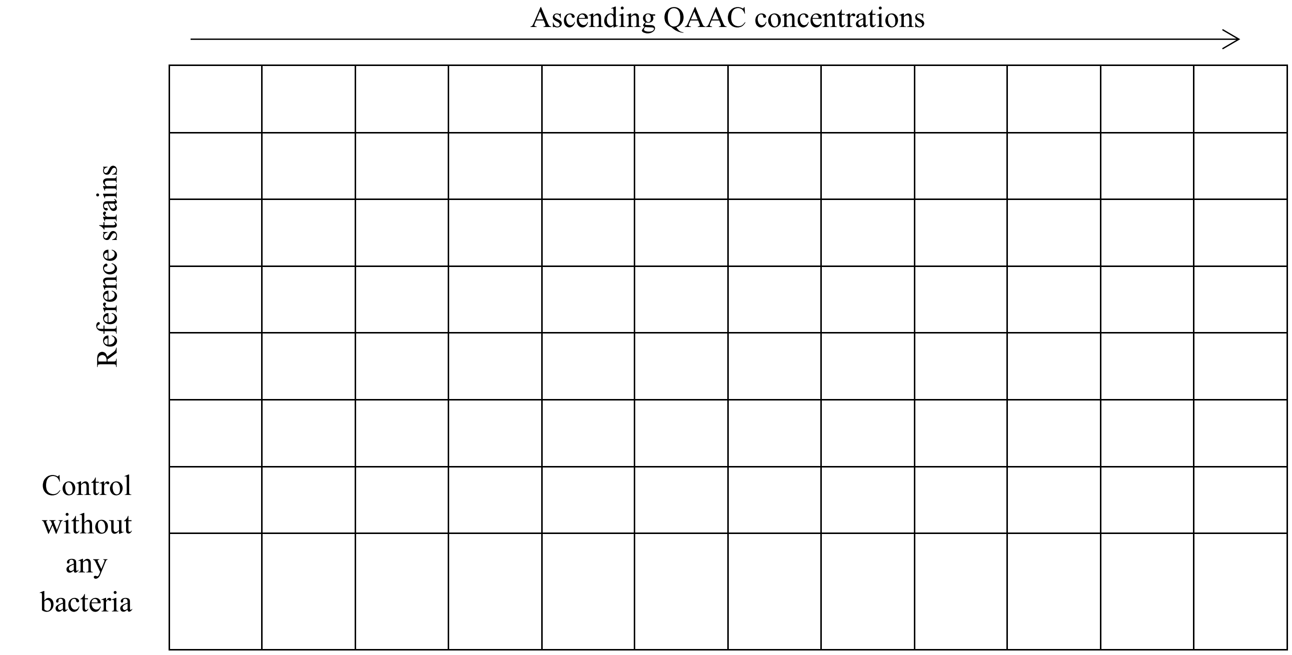

Table S6 Schematic structure of the 96-Well Plates as used in clay concentration test for MIC of *E. coli* ConF4 to QAAC (BAC-C12 and DADMAC-C10)

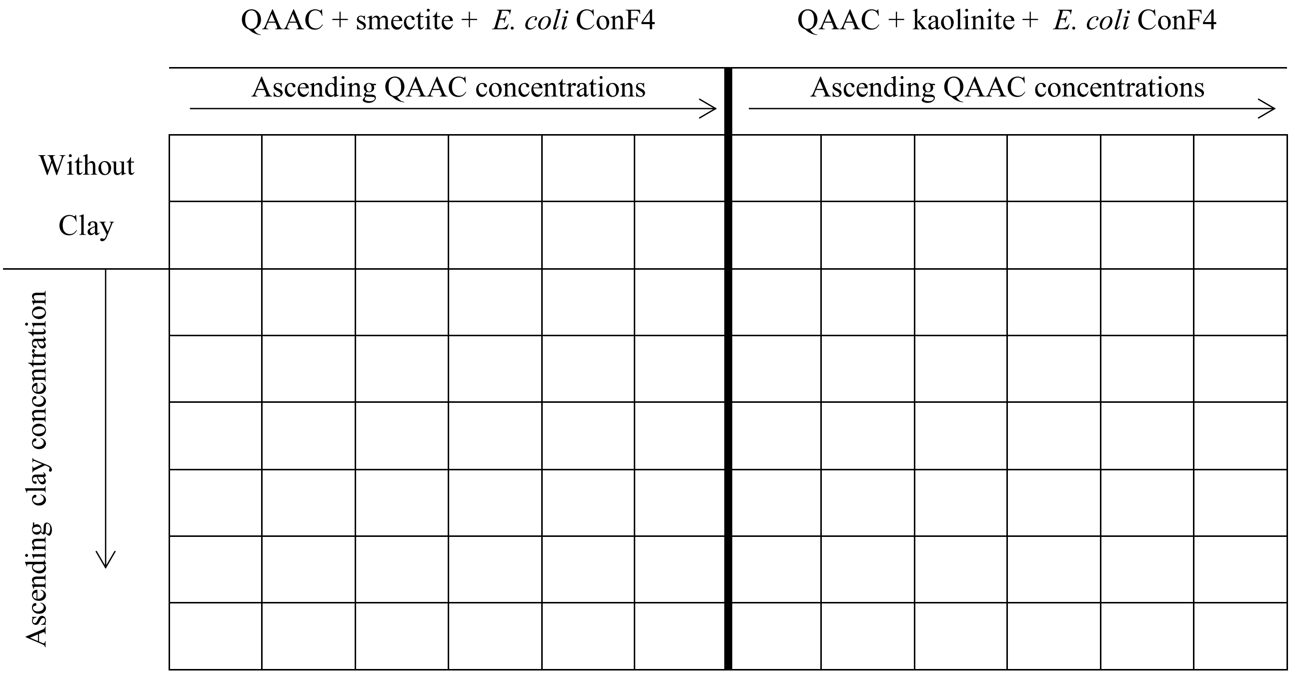

Table S7 Schematic structure of the 96-Well Plates as used in the final tests to determine MIC for *E. coli* ESBL37B15_13_1E, P. *fluorescens* DSM 50090^T, and *E. coli* ConF4 under influence of BAC-C12 and DADMAC-C10 and clay minerals

Without clay						Smectite or kaolinite						
0	6.25	12.5	25	50	100	0	6.25	12.5	25	50	100	BAC µg mL^{-1}
												E. coli ESBL37B15_13_1E
												P. fluorescens DSM 50090^T
												E. coli ConF4
												Control (without MO)

Table S8 MS/MS Instrument Parameters for positive ion multiple reaction-monitoring mode and ESI.

	Ion Transitions	Con Voltage [V]	Collision Energy [V]
DADMAC-C10	326.4 > 186.2	50	29
BAC-C12	304.2 > 90.6	40	25
MS/MS Parameters		Capillary [kV] = 3.50<br>Cone [V] = 43<br>Source Temperature [°C] = 130<br>Desolvation Temperatuer [°C] = 450<br>Desolvation Gas Flow [L h^{-1}] = 600<br>Cone Gas Flow [L h^{-1}] = 50<br>Collision Gas = Argon	
